# Endofungal bacteria as hidden facilitators of biotic interactions

**DOI:** 10.1093/ismejo/wraf128

**Published:** 2025-06-24

**Authors:** Ingrid Richter, Hannah Büttner, Christian Hertweck

**Affiliations:** Department of Biomolecular Chemistry, Leibniz Institute for Natural Product Research and Infection Biology (Leibniz-HKI), 07745 Jena, Thuringia, Germany; Department of Biomolecular Chemistry, Leibniz Institute for Natural Product Research and Infection Biology (Leibniz-HKI), 07745 Jena, Thuringia, Germany; Department of Biomolecular Chemistry, Leibniz Institute for Natural Product Research and Infection Biology (Leibniz-HKI), 07745 Jena, Thuringia, Germany; Faculty of Biological Sciences, Friedrich Schiller University Jena, 07743 Jena, Thuringia, Germany; Cluster of Excellence Balance of the Microverse, Friedrich Schiller University Jena, 07745 Jena, Thuringia, Germany

**Keywords:** bacterial-fungal interactions, disease control, ecosystem management, endosymbiosis, microbial ecology, pathogenicity, trophic interactions

## Abstract

Fungi play pivotal roles in ecology and human health, driving nutrient cycling, supporting antibiotic production, and posing threats through toxin production. Less well-recognized, however, is their ability to harbour endosymbiotic bacteria. Advances in genomics and microscopy have revealed the prevalence of endofungal bacteria across diverse fungal phyla, though their functions are primarily inferred from genomic and transcriptomic studies. Recent functional research has begun to shed light on their influence on fungal pathogenicity, physiology, and ecology. These findings raise fundamental questions about the establishment and benefits of bacterial-fungal endosymbiosis, as well as the role of endosymbionts in mediating fungal interactions with other organisms.

This review provides an in-depth analysis of the molecular mechanisms involved in the establishment and persistence of these symbioses. It also summarizes the current understanding of how endofungal bacteria impact fungal interactions with other organisms. For instance, endofungal bacteria contribute to the beneficial effects of fungi on plant health and fitness, protect fungal hosts from fungivorous predators, and enhance fungal virulence against plants, animals, and humans. These discoveries highlight the need for holistic investigations into bacterial-fungal endosymbiosis to fully understand their role in natural ecosystems. A deeper understanding of these multipartite partnerships offers exciting opportunities to improve ecosystem management, food safety, disease control, and crop productivity.

## Graphical Abstract

**Graphical Abstract:**
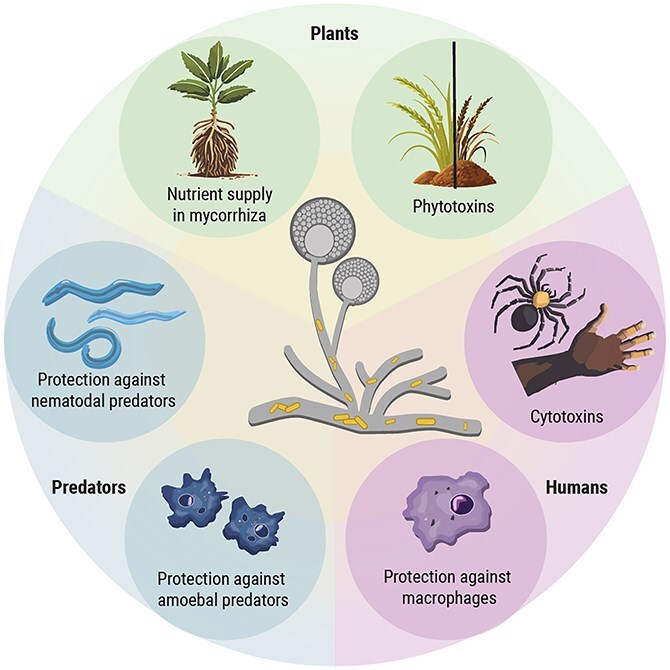

## Introduction

Members of the eukaryotic kingdom Fungi occupy a unique place in human culture, valued for their essential roles in food production, human health, and ecology. They help create delicacies such as cheese, add flavour and nutrients to our diets, and produce life-saving antibiotics. Moulds also play a critical role in healthy soils, breaking down xenobiotics, cycling minerals and organic matter, and supporting plant fitness [1–3]. In contrast, some fungi are feared for their ability to produce potent toxins that spoil food, damage agricultural crops, and cause serious diseases in plants, animals, and humans [4]. A large body of knowledge has been built up about the metabolic capabilities of moulds and the roles of specialized metabolites as toxins, pathogenicity factors, and protective agents. Given their profound impact on health, society, and the environment, it is striking that, until recently, it was largely overlooked that moulds can host endosymbiotic bacteria that can complement the biosynthetic potential of the fungi. In fact, several notorious mycotoxins are produced by these endosymbionts and not by the fungal host. The discovery of microbes within microbes not only alludes to the origins of eukaryotic life but also reveals hidden players that are essential to diverse biotic interactions [5–7].

Thanks to recent advances in genome sequencing technologies and state-of-the-art microscopy, an increasing number of bacteria residing inside fungal cells are being identified. The potential roles of these endofungal bacteria (also referred to as “endohyphal bacteria” or “endobacteria”) have mostly been inferred from metagenomic and transcriptomic data, as discussed in several reviews [5–13]. Only in recent years has it become possible to combine genomics with functional studies to investigate the roles of endofungal bacteria. The fact that endofungal bacteria impact fungal pathogenicity, physiology, and ecology raises a number of fundamental questions. How did endosymbiotic relationships establish and stabilize? What are the gains for the individual partners? What roles do endosymbionts play in the interactions of fungi with the environment and organisms?

In this review, we discuss recent discoveries that clarify the molecular mechanisms involved in the establishment and maintenance of bacterial-fungal endosymbiosis. A particular focus is placed on how endofungal bacteria influence fungal interactions with additional interaction partners, providing novel insights into complex interkingdom interactions and their roles in ecosystem functioning. We argue that an integrated view of endofungal bacteria can deepen our understanding of microbial ecology, which may assist in developing targeted interventions against plant and human diseases, enhancing crop yields and plant health, and ensuring food safety.

## Discovery and occurrence of endofungal bacteria

The first discovery of bacteria-like organisms (BLO) inside of fungi was made with electron microscopy in 1970 by Barbara Mosse [14]. While studying fine structures during *Endogone* spore development, she noticed that these self-replicating organisms are present in the cytoplasm of vegetative hyphae, where they form extensive colonies. In the following two decades, morphological studies provided additional evidence for the presence of BLOs in arbuscular mycorrhizal fungi such as *Glomus caledonium*, *Gigaspora margarita*, and *Jimgerdemannia flammicorona* (formerly known as *Endogone flammicorona*) [15–17]. In these pioneering studies, classification of fungi and their endobacteria relied exclusively on morphological characterization.

Despite the increased accessibility of molecular biological tools in the early 2000s, fungal endosymbionts were largely regarded as rare curiosities limited to a few mycorrhizal fungi, whose functions have remained unknown due to the inability to culture the bacterial symbionts. A study published in 2005 was the first to demonstrate that endofungal bacteria (*Mycetohabitans rhizoxinica*) play important ecological roles through the production of phytotoxic compounds [18, 19]. The isolation and cultivation of *M. rhizoxinica* outside its fungal host (*Rhizopus microsporus*) paved the way for genetic manipulation and fluorescent labelling of endosymbionts [20, 21]. In addition, the endosymbionts were successfully eliminated by antibiotic treatment, and the *Rhizopus*-*Mycetohabitans* symbiosis could be re-established through co-cultivation experiments [20], enabling the first direct functional studies of bacterial-fungal endosymbioses. In 2007, endosymbionts (*Candidatus* Glomeribacter gigasporarum) were successfully eliminated from spores of the arbuscular mycorrhizal fungus *G. margarita* BEG34 through repeated spore propagation [22]. This allowed comparisons between endosymbiont-containing and endosymbiont-free host genotypes, providing valuable functional insights into this bacterial–fungal interaction, despite the bacterial symbiont remaining unculturable [22]. These early functional studies were instrumental in transforming the field from descriptive observations to mechanistic understanding and laid the foundation for a growing body of research on endofungal bacteria.

**Figure 1:**
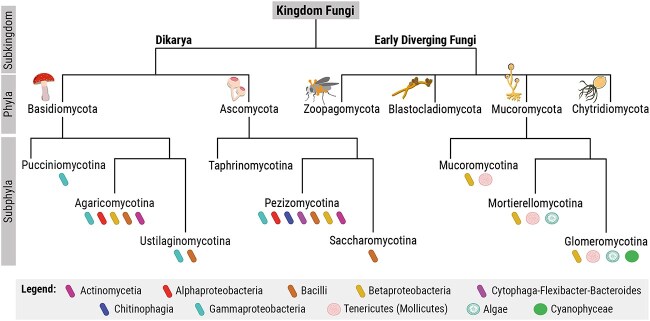
Representation of the diversity and distribution of endofungal bacteria. This schematic phylogenetic tree is not to scale; branch lengths do not represent evolutionary distances. The tree depicts one proposed variant of fungal phylogeny, showing only endofungal bacteria that are confirmed by fluorescence and/or electron microscopy.

With the rise of high-throughput sequencing technologies, the study of endofungal bacteria flourished, demonstrating that endobacteria are present in several fungal lineages ranging from Dikarya (Ascomycota and Basidiomycota) to early diverging fungi (Mucoromycota) (Fig. 1). Ascomycota primarily harbour endofungal bacteria belonging to the Gammaproteobacteria [23, 24], whereas Basidiomycota mainly contain Alphaproteobacteria [25–27] and in some instances Gammaproteobacteria [28–30]. The majority of fungal endobacteria identified to date have been found in members of the phylum Mucoromycota [31]. These endobacteria primarily belong to two taxa, Betaproteobacteria (*Burkholderia*-related) and Mollicutes (*Mycoplasma*-related), and are associated with fungi belonging to the three major classes of Mucoromycota: Glomeromycotina, Mortierellomycotina, and Mucoromycotina [32, 33]. These groups also include the best-characterized model systems for studying bacterial–fungal endosymbioses: (i) *Ca.* G. gigasporarum within Glomeromycotina fungi [34]; (ii) *Mycoavidus cysteinexigens* in association with Mortierellomycotina fungi [35, 36]; and (iii) *Mycetohabitans* spp. with *Rhizopus* spp. in the Mucoromycotina [18].

## Establishment and maintenance of bacterial-fungal endosymbioses

### Bacterial colonization of fungal hyphae

Although knowledge about the existence of endofungal bacteria has expanded rapidly over the past two decades, it still remains unclear how these endosymbiotic relationships were initially established. In general, endosymbiotic relationships often arise when the biochemical capabilities of an endosymbiont complement the host's limited metabolic functions. The most notable examples of such partnerships are the formation of mitochondria and chloroplasts from internalized prokaryotes, which brought respiration (mitochondria) and photosynthesis (chloroplasts) to eukaryotic cells, respectively [37]. Interestingly, the early interactions between eukaryotic cells and the bacterial ancestors of chloroplasts and mitochondria could have involved initial competition, invasion, or a pathogenic relationship before transitioning into a mutualistic, stable endosymbiosis [38–40]. Based on these observations, it is tempting to speculate that bacteria living inside fungi may have initially invaded their hosts as harmful pathogens. Over time, however, these organisms may have transitioned from a parasitic to a mutualistic relationship—or somewhere in between—adapting to life inside the host and providing essential functions or resources in exchange for protection and nutrients.

In order to infect and colonize a host, pathogenic bacteria inject or release effector molecules, toxins, and enzymes into host cells via specific secretion systems [41]. However, secretion systems are also widely used by non-pathogenic bacteria for communication, competition, and adaptation to different environments [41]. Interestingly, both pathogenic and mutualistic bacteria commonly utilize the Type II, III, IV, and VI secretion systems (T2SS, T3SS, T4SS, and T6SS) to either manipulate host cell processes, evade immune responses, and trigger cell death or to facilitate mutualistic interactions, respectively [41]. In fact, genes coding for these different secretion systems have been detected in multiple endofungal bacteria [35, 42–44]. Thus, the ability to secrete enzymes and effectors via secretion systems may have facilitated bacterial invasion into fungal cells, enabling a subsequent transition to a mutualistic lifestyle.

**Figure 2:**
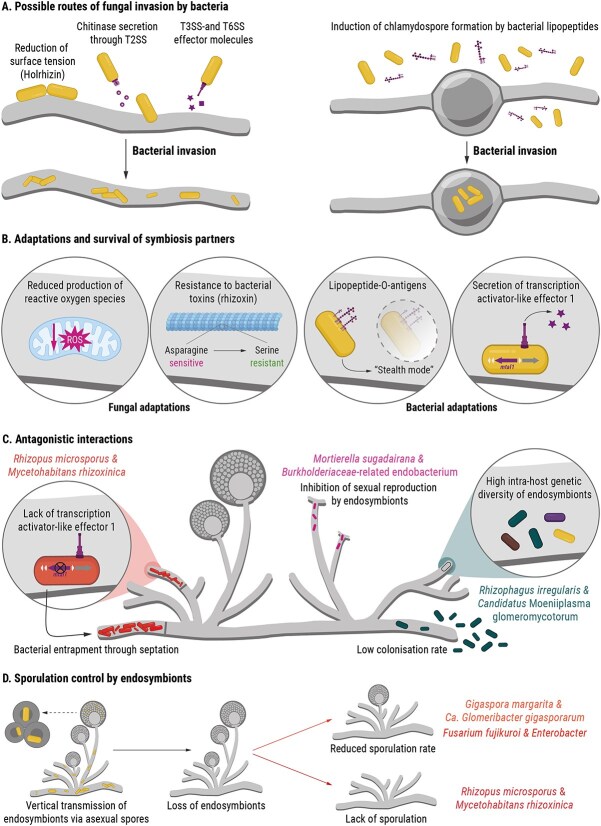
Known symbiosis factors that contribute to the establishment and maintenance of bacterial-fungal endosymbioses. (A) Model depicting the invasion of the fungus *Rhizopus microsporus* by *Mycetohabitans rhizoxinica* through the secretion of surfactants (lipopeptide holrhizin), chitinases, and effector molecules (left). Free-living bacteria (*Ralstonia* and *Bacillus*) invade chlamydospores of *Aspergillus nidulans* through the secretion of lipopeptides. (B) Adaptations and strategies that allow the establishment of a stable symbiosis between *R. microsporus* and *M. rhizoxinica*. (C) Examples of endofungal bacteria forming antagonistic relationships with their fungal hosts. (D) Schematic representation of the vertical transmission of endosymbionts via asexual spores. Loss of endosymbionts, either naturally or through experimental antibiotic treatment, leads to reduced sporulation in *Fusarium fujikuroi* and *Gigaspora margarita*, and complete loss of sporulation in *Rhizopus microsporus*.

The process of how bacteria invade fungal hyphae has been best explored in the symbiosis between the Mucoromycota fungus *Rhizopus microsporus* and its bacterial endosymbiont, *Mycetohabitans* rhizoxinica [18]. The ability to culture both symbiosis partners separately has made the *Rhizopus*-*Mycetohabitans* pairing a model system to study the molecular basis of bacterial-fungal endosymbiosis. Before making physical contact, genes encoding a T3SS machinery, candidate T3SS effector proteins, and a bacterial chitinase are upregulated in *M. rhizoxinica* [45]. T3SS are needle-like transporters that span the bacterial membranes and induce the secretion of effector proteins after contact with the fungal host cells [41]. Functional studies had previously shown that a T3SS is essential for bacterial entry into fungal hyphae [46], but the corresponding T3SS effectors have remained elusive (Fig. 2A). Bacterial attachment to the fungal hyphae is aided by a bacterially-produced linear lipopeptide (holrhizin A) that reduces surface tension [47] (Fig. 2A). This leads to altered biofilm formation and cell motility, which promote physical contact and therefore colonization [47]. Interestingly, free-living bacteria that do not form obligate symbioses with a host also utilize lipopeptides to facilitate fungal colonization [48, 49]. For example, the bacterial plant pathogen *Ralstonia solanacearum* secretes the lipopeptide ralsolamycin, which induces chlamydospore formation in the Ascomycota fungus *Aspergillus flavus* (Fig. 2A). The lipopeptide likely increases cell permeability, thus allowing *R. solanacearum* to colonize chlamydospores and subsequently adopting an endofungal lifestyle [48]. Such lipopeptides may also increase the permeability of the fungal cell membrane, thus promoting bacterial translocation across the fungal cell membrane. Bacterial entry through alteration of membrane permeability has been reported for the facultative endosymbiosis between the Mucoromycota fungus *Mucor irregularis* and the Gammaproteobacterium *Serratia marcescens* [50].

In general, a functional T2SS is considered essential for host invasion [51], which is supported by the fact that the majority of endofungal bacteria analysed to date harbour T2SS genes [44]. For example, facultative endofungal bacteria from diverse Ascomycota have the genetic potential to produce a T2SS [44]. The obligate endofungal bacterium *M. rhizoxinica* secretes chitin-binding proteins and chitinolytic enzymes via the T2SS [52] (Fig. 2A). The fungal host facilitates symbiont entry by upregulating genes involved in cell wall biosynthesis and cytoskeletal rearrangements [45]. Such changes in gene expression have also been observed in other bacterial-fungal symbioses. In the facultative symbiosis between *Pestalotiopsis* sp. (Ascomycota) and endofungal *Luteibacter* sp. (Gammaproteobacteria), cell wall- and membrane-associated genes are upregulated in the fungal host [53]. Genes encoding a T6SS machinery were also upregulated in *Luteibacter* sp. during early establishment of the symbiosis [53] (Fig. 2A). Both T2SS and T3SS as well as host cell wall remodelling have also been shown to be important for host colonization by *Ca.* Glomeribacter gigasporarum, an endosymbiont of *G. margarita* [54, 55]. These findings suggest a common strategy of bacterial invasion into fungi.

To better understand bacterial entry into fungal hyphae, a process that is often unpredictable in time and location, a laser beam transformation technique was developed for the controlled introduction of bacteria into fungi [20]. Focusing a laser beam on the fungal cytoplasm of *R*. *microsporus* resulted in the rapid uptake of *M. rhizoxinica* at a specific site by osmotic pressure [20]. More recently, a fluidic force microscopy approach was developed to inject bacterial cells directly into fungal hyphae to create artificial endosymbioses [56]. Adaptations in both partners were monitored over multiple rounds of adaptive laboratory evolution. Genomic adaptations occurred only in the fungal host, but not in the endosymbiont [56].These studies provide a foundation for further research into the molecular details of how endosymbiosis is established and maintained after bacterial entry.

### Fungal and bacterial adaptations enabling symbioses persistence

Once bacteria have successfully invaded fungal hyphae, both partners face potentially lethal challenges. Invading bacteria are confronted with the fungal innate immune system, which consists of components analogous to animal and plant immune mechanisms [45]. The molecular details of fungal immune responses are largely unknown and it remains to be tested whether fungi can discriminate between bacterial invaders and potential symbionts [6]. However, initial insights have been provided by transcriptomics, which revealed that *R. microsporus* aids *M. rhizoxinica* colonization by quenching reactive oxygen species (ROS) [45] (Fig. 2B), a key element of the non-host immune response [45]. The release of ROS is a common strategy used by plants and animals to fend off bacteria perceived as antagonists [57]. In addition, *R. microsporus* accommodates *M. rhizoxinica* through changes in the fungal lipid metabolism mediated by diacylglycerol kinases [58].

In order to persist within its fungal host, *M. rhizoxinica* produces a specialized bacterial lipopolysaccharide structure (Fig. 2B). Decorating its outer membrane with a d-galactofuranose O-antigen apparently allows the endobacterium to live in “stealth mode” possibly by evading recognition from as-yet-unidentified fungal immune receptors [59]. In addition, intracellular survival of *M. rhizoxinica* is promoted by transcription activator-like effector 1 (MTAL1) that is secreted by the T3SS (Fig. 2B). Using a combination of microfluidics and high-resolution live imaging, it was shown that the absence of MTAL1 induces the biogenesis of septa in *R. microsporus*, leading to hyphal trapping of endobacteria and subsequent death of MTAL1-deficient *M. rhizoxinica* [60] (Fig. 2C). This suggests that *R. microsporus* perceives *M. rhizoxinica* as a pathogen rather than an endosymbiont in the absence of MTAL1. Bioinformatic analysis identified the coding sequence of a membrane-bound transporter as a potential MTAL1 target in the *R. microsporus* genome [61]. However, experimental studies are needed to provide evidence for promoter binding, especially as the majority of matching target sequences fall within the 5′ untranslated region of genes encoding DNA-associated proteins or within assembly gaps [61]. A protective response by septation has also been observed in *R. microsporus* responding to injected *Escherichia coli* cells [56]. *E. coli* proliferated rapidly and the dividing cells stayed together. This resulted in the formation of “*E. coli* clumps”, and *R. microsporus* produced septa around these densely populated areas [56], suggesting that the fungus recognizes non-symbiotic bacteria and initiates a defense response to physically contain intruders. These results highlight the “sliding scale” nature of bacterial-fungal endosymbiosis, where symbionts can be perceived as mutualists or parasites depending on the context [62].

The fungal host, in contrast, is confronted with toxic metabolites produced by the bacteria. For example, *M. rhizoxinica* carries the genetic inventory to produce a variety of toxic secondary metabolites [63, 64]. The most infamous example is rhizoxin, a potent antimitotic toxin that causes blight disease in rice seedlings and is active against a range of eukaryotic cells, including fungi [18, 65, 66]. One of the key factors in establishing a durable symbiotic relationship is that *R. microsporus* (and other Mucorales fungi) acquired resistance to rhizoxin by exchanging asparagine for serine at amino acid position 100 in the β-tubulin chain [66] (Fig. 2B). Similarly, genomic adaptations in the fungal host to accommodate bacterial endosymbionts were recently observed in an adaptive laboratory evolution experiment. *M. rhizoxinica* was directly injected into a non-host *R. microsporus* strain using fluidic force microscopy [56]. These bacteria were able to propagate and disperse within the fungal hyphae without apparent harm to the fungus. In addition, *M. rhizoxinica* is translocated into non-host *R. microsporus* spores [56]. The new symbiotic pair was subjected to several rounds of adaptive laboratory evolution during which host fitness was assessed by measuring both the number of spores containing endosymbionts and their germination success. By the end of the experiment, spores with endosymbionts had similar germination success to those without, indicating improved fitness through adaptation. This increased fitness appeared to result from genomic changes in the fungus, as no significant adaptations were observed in the endosymbiont. Bioinformatic analysis suggests that the functions of the adapted genes may be related to endocytosis, translation, and transcription. Although the exact mechanisms remain unclear, the genetic changes in the fungal population correlate with the increased fitness and stability of the evolved endosymbiosis—defined as the ability to produce viable offspring containing endosymbionts [56].

To ensure the persistence of bacterial–fungal endosymbioses, endofungal bacteria must be passed on to the offspring through either asexual or sexual spores (Fig. 2D). Vertical transmission of endosymbionts via asexual spores has been reported in each of the three Mucoromycota subphyla: Glomeromycotina [67–69]*,* Mortierellomycotina [70]*,* and Mucoromycotina [20, 71]. In the Mucoromycotina fungus *R. microsporus*, *Mycetohabitans* bacteria control asexual (vegetative) sporulation [20], but only enhance the mating success of compatible strains during sexual reproduction [71]. When treated with antibiotics to remove its endosymbionts, *R. microsporus* loses its ability to reproduce vegetatively (Fig. 2D). This reliance on *M. rhizoxinica* for sporulation ensures the persistence of the symbiosis over time [20, 71]. Packaging of endosymbionts into airborne fungal spores also facilitates dissemination of symbiotic *Rhizopus* strains in the environment through wind and insects [72]. Indeed, the *Rhizopus*-*Mycetohabitans* alliance is globally distributed across all five continents inhabiting a variety of niches ranging from temperate and arid soils to human tissue [73–75]. A number of studies have identified bacterial factors that are important for *R. microsporus* sporulation, including bacterially produced secondary metabolites [64, 76], the deazaflavin metabolite F_O_^77^, and T3SS-associated transcription activator-like effectors [61], which are widely distributed among endofungal *Mycetohabitans* strains [61, 77]. Vertical transmission of endosymbionts via asexual spores has also been reported in the Glomeromycotina [67, 69]. Spores of these arbuscular mycorrhizal fungi commonly contain *Candidatus* Moeniiplasma glomeromycotorum, and in rare cases, *Ca.* Glomeribacter gigasporarum [78, 79]. Additionally, a recent study revealed unexpectedly high bacterial diversity in spores of arbuscular mycorrhizal fungi, with endobacteria belonging to 10 different phyla [69]. Interestingly, the diversity of these additional endobacteria was found to be negatively correlated with the presence of *Ca.* Moeniiplasma glomeromycotorum, suggesting that it may outcompete other endosymbionts [69]. Despite this, the role of *Ca.* Moeniiplasma glomeromycotorum in the biology of arbuscular mycorrhizal fungi remains experimentally untested. The less abundant *Ca.* Glomeribacter gigasporarum has been shown to increase the sporulation rate of its host *G. margarita* [54] (Fig. 2D). These endofungal bacteria do not only improve the sporulation success of their host [54], but also influence spore morphology and fungal pre-symbiotic growth. The absence of *Ca.* Glomeribacter gigasporarum affects the cytoplasmic organization, vacuole morphology, cell wall organization, lipid bodies, and pigment granules of fungal spores. These changes result in delays in the growth of germinating mycelium, which may negatively influence the ecological fitness of the fungus [22]. Endosymbiont-dependent vegetative reproduction has also been observed in Basidiomycota and Ascomycota. For example, the fungal mutualist *Serendipita indica* (formerly known as *Piriformospora indica*) requires its endosymbiont *Rhizobium radiobacter* (formerly known as *Agrobacterium radiobacter*) to produce vegetative chlamydospores [80]. In the plant-pathogenic fungus *Fusarium fujikuroi* a loss of *Enterobacter* symbionts leads to reduced production of macroconidia [81] (Fig. 2D).

Compared to transmission via asexual spores, less is known about how endofungal bacteria are transmitted through sexual spores. Endosymbionts of the Mucoromycota fungus *Mortierella sugadairana* inhibit sexual reproduction through as yet unknown mechanisms [70], suggesting that housing endosymbionts may impose fitness costs on the fungal host if these bacteria behave more like pathogens than mutualists. In the *Rhizopus–Mycetohabitans* symbiosis, endosymbiont-free fungi produce fewer sexual zygospores than symbiotic *R. microsporus* [71]. By assessing global gene expression patterns, it was suggested that the endofungal bacteria control the transcription of the fungal *ras2* gene, which encodes a GTPase central to fungal reproductive development [71]. Thus, in contrast to asexual sporulation, mating of symbiotic *R. microsporus* is not fully controlled by endofungal bacteria. Adding further complexity, one *R. microsporus* strain not only forms a symbiosis with bacteria but also with two viral members of the genus *Narnavirus* (RmNV-20S and RmNV-23S) [82]. It was shown that together with *Mycetohabitans*, narnaviruses are required for successful sexual reproduction [82]. This ability of endofungal bacteria to control their own transmission supports the hypothesis that these symbionts originated from parasitic ancestors that gradually transitioned to mutualists. This is in line with evolutionary theory suggesting that many heritable mutualisms arise from initially antagonistic interactions [83].

### Are endofungal bacteria mutualists or parasites?

It is conceivable that endofungal bacteria were originally pathogens, as they often encode an arsenal of genes related to a pathogenic lifestyle. For example, *Ca.* Glomeribacter gigasporarum, despite its notably small genome (1.72 Mb), contains genomic features characteristic of both symbiotic bacteria (e.g. genome reduction, host-nutritional dependence) and pathogenic bacteria (e.g. the presence of a T2SS, T3SS, and T4SS), suggesting a transitional state between the two lifestyles [55]. Such transitions from mutualism to antagonism have also been reported for the obligate *Rhizopus*-*Mycetohabitans* symbiosis, where inhibition of diacylglycerol kinases alters the fungal lipid profile in a way that causes a shift from mutualism to antagonism [58]. This shift is not surprising, considering that *M. rhizoxinica* was probably initially a parasite before becoming a mutualist [58, 66, 73]. Indeed, the highly reduced *M. rhizoxinica* genome (3.75 Mb) encodes genes for six different secretion systems (types 1–6), putative effector molecules, and a higher number of transposons and virulence-related genes than other members of *Burkholderia sensu lato* [63, 64]. Therefore, it is conceivable that the absence of MTAL1 proteins [60] or diacylglycerol kinases [58] could cause a reversion from a mutualistic to a parasitic lifestyle. These findings underscore the dynamic nature of bacterial–fungal endosymbioses, which can range from mutualistic to parasitic depending on the environmental or physiological context [62], ultimately resulting in a partnership that can be beneficial or detrimental to the individual partners.

Several relationships between endobacteria and their fungal host are characterized as purely antagonistic [70]. A well-studied case is *Ca.* Moeniiplasma glomeromycotorum, which may act as an antagonist to its host [84, 85]. For example, the sporulation rate of the Glomeromycota fungus *Rhizophagus irregularis* (formerly known as *Glomus intraradices*) is greatly reduced in the presence of endosymbionts (*Ca.* Moeniiplasma glomeromycotorum) [86]. These *Mycoplasma*-related endosymbionts also show a low host colonization rate compared to other non-endosymbiont-containing *R. irregularis* strains [87] (Fig. 2C), suggesting that *Ca.* Moeniiplasma glomeromycotorum are parasites rather than mutualists. Furthermore, only one out of 58 *R. irregularis* isolates was found to harbor endosymbionts supporting the notion that these bacteria are rather parasites than facultative mutualists [88]. This is supported by molecular evolutionary patterns, extrapolated from 16S rRNA gene phylogenies, that revealed high intra-host genetic diversity of endosymbionts within individual *R. irregularis* hosts [85] (Fig. 2C). High genetic variation within hosts is a common feature of parasites as they maintain genetic diversity through recombination and horizontal transmission. Interestingly, high population heterogeneity of *Ca.* Moeniplasma glomeromycotorum within individual Glomeromycota hosts appears to be common [78], with up to 50 operational taxonomic units detected in a single arbuscular mycorrhizal fungus spore [69]. This high genetic diversity is unusual for mutualists that are transmitted vertically, as maintaining genetic diversity within a single host may fuel symbiont competition, increase metabolic burden on the host, and facilitate horizontal transmission to a new host for symbiont survival [89]. Despite these clear characteristics of parasites, *Ca.* Moeniiplasma glomeromycotorum are the most abundant endosymbionts in arbuscular mycorrhizal fungi [78, 79]. This raises the question of why *Ca.* Moeniiplasma glomeromycotorum are maintained within populations of arbuscular mycorrhizal fungi if they do not provide any benefits to the host, as one would expect selection to eliminate such parasites from the population [90]. Although the ecological role of maintaining a parasitic relationship remains largely unknown, it has been recently suggested that the “parasitic” *Ca.* Moeniiplasma glomeromycotorum may outcompete other potentially detrimental endobacteria [69]. A deep sequencing approach has revealed a high diversity of endobacteria within spores of arbuscular mycorrhizal fungi. However, spores with a high *Ca.* Moeniiplasma glomeromycotorum diversity contain less diverse communities of other endobacteria [69], suggesting that *Ca.* Moeniiplasma glomeromycotorum act as conditional mutualists. In a conditional mutualism, the benefits provided by the symbiont to the host depend on specific conditions, such as ecological context, the symbiont’s life history stage, and its population size [91]. This suggests that by tolerating a highly diverse population of *Ca.* Moeniiplasma glomeromycotorum, the fungal host may be able to defend itself against other potentially harmful endobacteria. Though this hypothesis requires experimental confirmation, two analogous examples exist in which mutualists of *R. microsporus* and *Podila verticillata* protect their hosts from soil-dwelling micropredators [92, 93].

## Balance of give and take in bacterial-fungal symbioses

### What do endofungal bacteria gain from the symbiosis?

The consequence of an obligate endofungal lifestyle is that the symbiotic bacteria are dependent on host-derived nutrients—a host-dependent interaction that borders on parasitism. Although functional studies are lacking for most systems, advances in sequencing technologies have enabled the elucidation of many endosymbiont genomes, providing valuable insights into the nutrient requirements of endofungal bacteria. Phylogenetically diverse Gram-negative bacteria living as facultative or obligate endosymbionts benefit from the uptake of nutrients from the fungal host.

**Figure 3:**
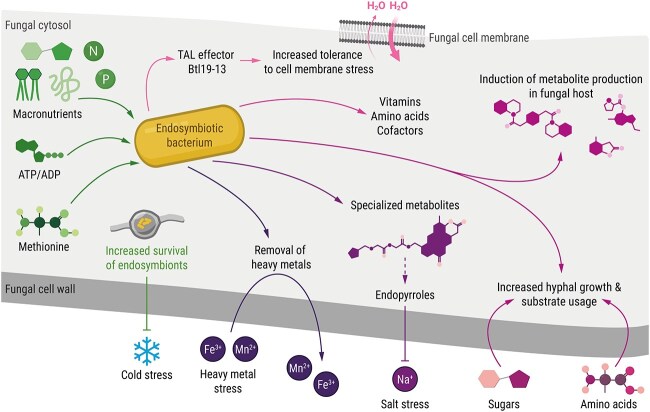
Illustration highlighting give and take in bacterial-fungal endosymbioses. Benefits for the endosymbiont are depicted in green, the gains for the fungal host are shown in pink/purple. This figure presents an overview of various known modes of interaction derived from different model systems.

Genomes of obligate endosymbionts have adapted to this intracellular nutrient supply, resulting in extensive gene loss, particularly in pathways related to basic metabolism. Using a comparative genomic approach, it was shown that *Mycoavidus* endosymbionts of Mortierellaceae fungi lack entire biosynthetic pathways, such as cysteine biosynthesis [94]. In addition, *Mycoavidus* endosymbionts generally exhibit limited β-oxidation capabilities compared to their free-living relatives [94]. The obligate endofungal bacterium *Ca.* Glomeribacter gigasporarum is strictly dependent on its host, *G. margarita*, for the supply of nutrients such as carbon, nitrogen, and phosphorus [55] (Fig. 3). In the *Rhizopus*–*Mycetohabitans* symbiosis, genomic data suggested that *M. rhizoxinica* consumes host metabolites such as citrate, malate, glycerol, and ethanol [63]. The importance of glycerol in the symbiotic relationship was recently confirmed using a genome-scale metabolic model constructed from genomic data of *R. microsporus*, *Mycetohabitans*, and the narnaviruses RmNV-20S and RmNV-23S^96^. Simulations of metabolic fluxes revealed that *Mycetohabitans* bacteria can efficiently consume glycerol without compromising fungal growth [95]. In addition, an increase in the host's lipid and nucleotide metabolism appears to be crucial for the functionality of this fungal–bacterial–viral symbiosis [95]. *Mycetohabitans* also harbours genes responsible for the import of branched-chain and aromatic amino acids as well as ATP/ADP antiporters suggesting that *M. rhizoxinica* may directly withdraw energy equivalents from the host cytosol [63] (Fig. 3). Such specialized antiporters are typically found in obligate intracellular pathogens such as *Rickettsia* [96] or the plant pathogen *Xylella fastidiosa* [97], again illustrating the fine line between parasitic and mutualistic lifestyles.

In facultative endosymbioses, internalized bacteria gain a nutritional advantage (glycogen, lipids) over non-invaders, thereby increasing bacterial fitness under starvation [48, 49]. In the *Pestalotiopsis*-*Luteibacter* symbiosis, dual-transcriptome sequencing data showed that all genes related to methionine metabolism are downregulated in the bacterial endosymbiont. This downregulation by *Luteibacter* is complemented by an upregulation of methionine metabolism in the fungal host *Pestalotiopsis*, suggesting that endofungal *Luteibacter* obtain organic sulphur through methionine acquisition from the host [53] (Fig. 3). In addition to nutrient supply, residence within fungal hyphae offers a safe niche, transport, and protection from abiotic stresses. When *R. solanacearum* colonizes chlamydospores of *A. flavus* [48], the bacteria exhibit increased cold survival compared to their free-living counterparts [49]. Thus, colonization of fungi provides a survival advantage to bacteria under abiotic stress (Fig. 3).

### What does the fungal host gain from the symbiosis?

The fungal partner may also benefit from its endosymbiont to cope with nutrient limitations and environmental stressors. For example, *Bacillus tequilensis* ITD-UANL-01, an endosymbiont of the Ascomycota fungus *Kluyveromyces marxianus*, has nitrogen-fixing capabilities and contributes to *K. marxianus* survival under nitrogen-limiting conditions by supplying usable nitrogen [98]. Fixation of atmospheric nitrogen by endofungal bacteria has also been reported in the symbiosis between the maize pathogenic fungus *Ustilago maydis* and the intracellular bacterium *Bacillus pumilus* [28]. In the *Rhizopus*-*Mycetohabitans* symbiosis, a bacterial transcription activator-like effector (Btl19–13) contributes to the host’s tolerance to cell membrane stress induced by sodium dodecyl sulphate [99] (Fig. 3). *M. rhizoxinica* produces non-ribosomal peptides (endopyrroles) exclusively in symbiosis with *R. microsporus* and mainly under salt stress [100] (Fig. 3). Although this suggests a potential role in increased salt stress tolerance of the fungal host, the impact of endopyrroles on the host remains to be elucidated. A recent study investigated the effect of endofungal bacteria on the resistance and uptake of essential and non-essential metals by the fungal host. Endosymbionts of *Benniella erionia* (Mortierellomycotina) increase heavy metal resistance (such as Mn^2+^) and Fe^2+^ removal compared to endosymbiont-free *B. erionia* [101] (Fig. 3). In addition, the number of endosymbionts in the fungal hyphae, as measured by the relative abundance of the 16S rRNA gene, increased with higher concentrations of essential metals such as Cr^2+^, Cu^2+^, Ca^2+^, and Mn^2+^, compared to control strains grown in metal-free media. This suggests a dependence of the fungal host on endobacteria for metal removal [101].

In many cases, endofungal bacteria contribute positively to fungal growth. For example, mycelial growth of the seed-associated fungus *Fusarium keratoplasticum* (Ascomycota) is promoted by endofungal bacteria (*Chitinophaga* sp.), presumably through better substrate utilization by the fungus-bacterium pair [102] (Fig. 3). *S. indica* requires its endosymbiont *R. radiobacter* for normal, healthy growth [80], and *G. margarita* depends on its obligate endofungal bacterium *Ca.* Glomeribacter gigasporarum for normal hyphal elongation and branching [22]. However, in some cases, endofungal bacteria may negatively impact biomass production. One possible explanation for the observed increase in biomass production in the absence of endosymbionts is the nutritional cost that many endosymbionts impose on their host. This cost may limit nutrient availability, potentially reducing fungal growth [103].

Despite disadvantages for their host, endofungal bacteria may also provide essential metabolites such as vitamins or cofactors, as is common for insect endosymbionts [104]. For example, in the *Ca.* Glomeribacter gigasporarum–*G. maragarita* symbiosis, the endobacteria synthesize vitamin B12 (Fig. 3). The genome of *M. rhizoxinica* encodes efflux transporters for basic amino acids and cysteine as well as genes involved in the biosynthesis of essential cofactors (e.g. pyridoxin, heme, flavin, biotin, thiamine) and the rare cofactor 3PG-F_420_ [63, 105] (Fig. 3). Gene expression analysis and metabolic profiling revealed that the biosynthesis of 3PG-F_420_, and its precursor F_O_, is particularly pronounced during symbiotic growth. Functional studies revealed that sporulation of *R. microsporus* is likely regulated by secretion of F_O_ into the host cytosol [106]. F_O_ is detectable in the supernatant of pure *M. rhizoxinica* cultures [105, 107], indicating that it is a secreted vitamin or signal regulating host sporulation.

In addition to metabolites related to primary metabolism such as amino acids and vitamins, endofungal bacteria are capable of producing specialized metabolites and effector molecules that may provide benefits for the interacting partners (Fig. 3). *M. rhizoxinica*, the endosymbiont of *R. microsporus*, devotes 9% of its highly reduced genome to specialized metabolite production [63, 64]. Natural products produced by *M. rhizoxinica* include polyketides (rhizoxin, necroxime) [65, 108], various non-ribosomal peptides such as cyclopeptides (rhizonin and heptarhizin, also known as rhizomide) [76, 109, 110], depsipeptides (habitasporin, endopyrole) [64, 100], the linear lipopeptide holrhizin, which has been shown to facilitate invasion of *R. microsporus* hyphae [47], and ribosomally produced and post-translationally modified peptides (RiPPs) such as lasso peptides (burhizin-23, mycetohabin-16, and mycetohabin-15) [111]. Comparative genomics of 26 *Mycoavidus* endosymbionts and 44 free-living bacterial relatives revealed biosynthetic gene clusters that are unique to *Mycoavidus* and related endosymbionts [94]. For example, two classes of non-ribosomal peptide synthases, predicted to produce the cytotoxic compounds rhizomides (also known as heptarhizin) [76] and luminmides, have no known analogs in free-living *Mycoavidus* relatives [94]. A staggering 12% of the genome of *Candidatus* Mycoavidus necroximicus, the endosymbiont of a *P. verticillata* strain, is occupied by biosynthetic gene clusters [92]. Yet, only necroximes and symbiosin have been identified as metabolites produced in this symbiosis [92, 112]. Endofungal bacteria not only possess the genetic inventory to produce specialized metabolites but are also able to induce metabolite production in the fungal host [81, 113] (Fig. 3). For example, *Enterobacter* symbionts induce fumonisin production in their fungal host *F. fujikuroi*, which contributes to increased virulence of *F. fujikuroi* [81]. Production of the phytohormone indole-3-acetic acid (IAA) is significantly enhanced in the foliar endophyte *Pestalotiopsis* aff. *neglecta* when an endofungal *Luteibacter* strain is present [113]. Both symbiotic partners rely on an l-tryptophan-dependent pathway for IAA synthesis and *Luteibacter* produces IAA exclusively under symbiotic conditions [113]. Although the role of many bacterially produced compounds remains unknown, a number of functional studies have recently revealed their role in mediating interactions of their fungal hosts with additional soil inhabiting organisms.

## Impact of bacterial-fungal endosymbioses on the environment and higher-order interactions

In addition to the intimate partnership of fungi and their endofungal bacteria, further interactions occur with other organisms in the various habitats where symbiont-harbouring fungi live. Although direct contact primarily happens between the fungus and other external organisms in the environment, bacterial symbionts inside the fungal cells have large impacts on the different interactions: endofungal bacteria can confer cytotoxicity against plants, animals, and humans through the production of bioactive natural products [11, 18, 92, 93, 108, 114] and influence the morphology or reproduction of the fungus [34]. Such changes may result in secondary effects on other interaction partners in the fungal environment. Although most studies of endosymbiont-harbouring fungi so far have focused only on the interactions between the bacterial symbiont and the fungal host, an increasing number of studies have examined the effects on additional interaction partners, shedding light on the role of endosymbionts in multipartite interactions and providing a more holistic understanding of the fungal habitats.

### Endofungal bacteria improve the nutritional supply in mycorrhizal interactions

**Figure 4:**
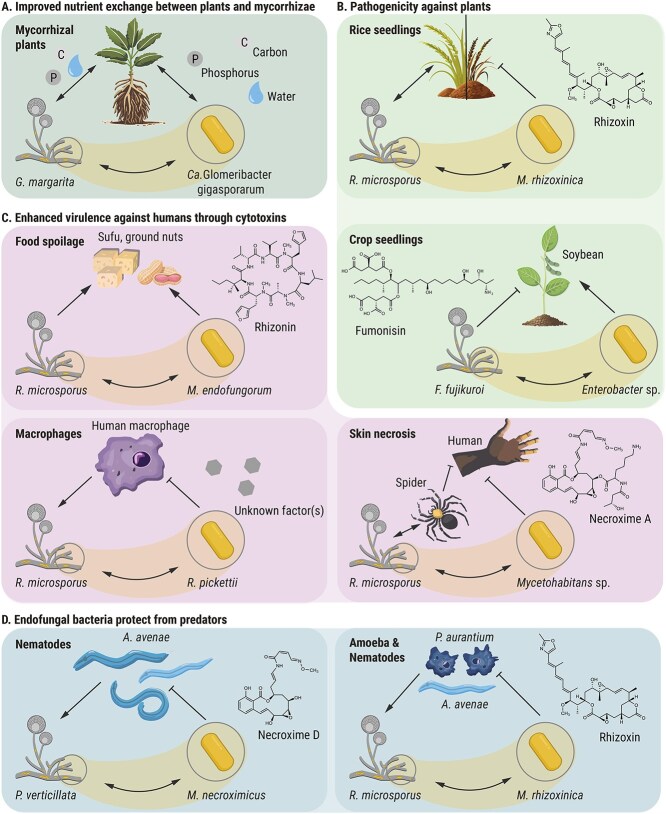
Tri- and multipartite interactions of endosymbiont-harboring fungi. (A) Interactions between fungi and endofungal bacteria with mycorrhizal plants have beneficial effects for all involved partners through an improved nutrition supply. (B) The presence of phytotoxin-producing endofungal bacteria confer pathogenicity to host fungi. (C) Bacterial toxins may enhance virulence of human-pathogenic fungi. (D) In another context, toxins produced by endobacteria may protect the fungal host form predators such as fungivorous nematodes and amoeba.

In recent years, the influence of endofungal bacteria on fungi in plant interactions has received considerable attention. Such associations of plants with fungi play crucial roles in terrestrial ecosystems. Up to 90% of all land plants form mutualistic mycorrhizal interactions with diverse fungal species [115]. The fungal hyphae extend the roots of the partner plants, thereby improving water, nitrate, and phosphate uptake [7]. The plants provide up to 20% of their fixed carbon to the fungal partners [115, 116] (Fig. 4A). However, endosymbiotic bacteria of endomycorrhiza-forming Glomeromycotina (arbuscular mycorrhizal fungi, AM fungi), but also ectomycorrhiza-forming Basidiomycetes have an influence on the symbiosis [8].

The beneficial effects of bacteria and endobacteria for the interaction between AM fungi and plants have been the subject of many studies and reviews [34, 117–119]. AM fungi grow inside the cortical cells of plant roots and form an arbuscule, a tree-like shaped structure which serves as the main exchange point between both mycorrhizal partners. In addition to the two “typical” mycorrhizal partners—fungus and plant, up to 88% of the involved Glomeromycotina fungi harbour diverse sets of endofungal bacteria [79]. The beneficial effects of these bacteria as additional symbionts include improved lipid metabolism of the fungus, control of fungal cell wall thickness, and increased nutrient uptake by the fungal host for the benefit of the plant symbiont [34, 54].

Ectomycorrhizal fungi are also supported by the presence of endofungal bacteria. These symbiotic mycorrhizal fungi colonize plant roots, but instead of penetrating plant cells, their hyphae surround root cells and form an interface for metabolite exchange known as the Hartig net [120]. The favourable effects of the improved nutrient exchange, commencing after ectomycorrhiza formation, are enhanced in mycorrhizas with endosymbiont-harbouring fungi [119]. In the case of pines (*Pinus massoniana* and *Pinus sylvestris*) some endofungal bacteria of the ectomycorrhiza-forming fungi *Suillus collinitus* and *Tylopilus neofelleus* increase the uptake of phosphorus by the fungus, thereby improving the supply of this vital nutrient to the trees [121, 122].

Besides improved nutrient supply, several endofungal bacteria of endophytic non-mycorrhizal fungi enhance the stress resistance of the interacting trees, such as in the case of the Mediterranean cypress *Cupressus sempervirens* [123]. It has been shown that one third of the associated endophytic ascomycotan fungi live in symbiosis with endofungal *Bacillus* or *Sphingomonas* bacteria, which influence the plant-fungal association [123]. The trees gain resistance to phytopathogenic antagonists in the presence of endophytic fungi harbouring endofungal bacteria [123].

The fitness of various higher plants living in symbiosis with the endophytic basidiomycete *S. indica* (see above, 3.1) is enhanced when the fungus harbours endosymbionts [80]. Barley, wheat, or *Arabidopsis thaliana* show higher growth rates and an improved systemic resistance to powdery mildew infection when living with fungal mycorrhiza partners containing high numbers of endofungal *R. radiobacter* bacteria [80]. Endofungal bacteria influence the growth of fruiting body-forming fungi, such as Basidiomycetes, regardless of whether the bacteria thrive inside the fungal hyphae or live inside the fruiting bodies. Taken together, endofungal bacteria can influence the establishment of ectomycorrhiza formation and thus indirectly the positive consequences of the plant-fungus symbiosis [124].

### Metabolites of bacterial endosymbionts cause pathogenicity against plants

In contrast to the beneficial effects that bacterial endosymbionts of mycorrhizal fungi have on plant growth and health, endofungal bacteria of *Rhizopus* and *Fusarium* spp. exert negative effects on plants. An infamous example is the bacterial production of phytotoxins of the rice seedling blight fungus *R. microsporus* [18] (Fig. 4B). The highly antimitotic agents, which target β-tubulin and act at nanomolar concentrations [66], are cooperatively biosynthesized by both symbiosis partners: the endobacteria (*M. rhizoxinica*) produce the polyketide backbone [21, 65, 125, 126], and the fungal host adds one epoxide ring to increase the phytotoxicity [19]. The toxin causes an abnormal swelling of rice seedling roots and, eventually, death of the plant. Both, the host and the endosymbionts, benefit from the release of nutrients. This toxin-producing alliance has an evolutionary advantage over related, symbiont-free saprotrophs lacking such powerful phytotoxins.

Endofungal bacteria may also play a role in crop diseases caused by *F. fujikuroi* [81]. This Ascomycete biosynthesizes fumonisins, which are highly toxic against humans and plants [127]. A recent study correlated the presence of endofungal *Enterobacter* bacteria in *F. fujikuroi* with higher toxin production and increased virulence [81] (Fig. 4B). Whereas the cured fungus has no significant effect on the growth of tested soybean seedlings, fungi containing endobacteria induce severe growth impairment with significant reductions in plant height and growth [81].

Panama disease, an infection of banana plants by *Fusarium oxysporum* species, is affected by bacterial symbionts of some fungal strains [128]. Whereas endosymbiotic *Izhakiella australiensis* bacteria have no effect on pathogenicity against banana plants, *Enterobacter* bacteria increase fungal virulence. Culture extracts of the symbiotic fungus cause leaf senescence and rhizome discoloration, more severely than culture extracts from cured fungi. Although the exact mode of action is still unclear, it was suggested that effectors such as fusaric acid could play a role in pathogenicity and enhance the virulence of the fungus [128].

### Impact of endofungal bacteria on the well-being of humans and animals

The presence of toxin-producing endofungal bacteria can also be a threat for humans. In South-East Asia, *Rhizopus* species are commonly used for the fermentation of food, in particular soybeans and soybean curd, to produce tempeh and sufu, respectively [129]. It is alarming that rhizoxin-producing endobacteria have been detected in a *R. microsporus* starter culture for sufu production [130], and various congeners of the potent antimitotic agent are produced in ample amounts under typical tempeh fermentation conditions [131]. In an effort to identify the agent that causes liver cancer in certain African tribes, a highly toxinogenic *R. microsporus* strain was isolated from mouldy peanuts in Mozambique [132]. From this strain two hepatotoxic cyclopeptides named rhizonin A and B were isolated, which proved to cause lethal liver lesion in animal experiments [133]. Although rhizonin was coined as "the first mycotoxin” reported from a fungus which belongs to the order Mucorales of the class Phycomycetes (lower fungi) [132], genetic, microbial, and chemical analyses revealed that the fungus is not the true producer of the toxin [109]. In fact, the fungal isolate harbours bacterial endosymbionts (*Mycetohabitans endofungorum*) [109], which assemble the toxin inolving a non-ribosomal peptide synthetase that incorporates unusual pharmacophoric furylalanine building blocks [109, 134] (Fig. 4C). Such building blocks are also found in endolides A and B, peptides biosynthesized by endofungal bacteria (*Burkholderia contaminans*) [135] of the marine fungus *Stachylidium bicolor* that lives in symbiosis with the marine sponge *Callyspongia* sp. cf. *C. flammea* [136]. Endolide A interacts with receptors of the vasopressin-type, whereas endolide B is selective for serotonin receptors [136].

Beyond food spoilage, endofungal bacteria have been implicated in the virulence of their fungal hosts to humans. Initially, there was no evidence for the involvement of endofungal bacteria in the pathogenesis of *R. microsporus* in humans or animals [137, 138]. However, in some severe cases of mucormycosis, the presence of bacterial endosymbionts in the causative *R. microsporus* fungi may influence the outcome of the infection [139, 140]. Recent data demonstrate a contribution of endosymbionts to fungal virulence in zebrafish and mouse models [114]. This protective effect is due to one or more secreted factors associated with *Ralstonia pickettii,* a bacterial endosymbiont of a clinical *R. microsporus* isolate (Fig. 4C) [114]. The antiphagocytic activity correlates with inhibition of phagocyte maturation, leading to pathogenicity in amoebae, macrophages, and animal models [114]. In addition, endobacteria show significant effects on the fungal stress response during macrophage confrontation and the protection of fungal spores from clearance by human macrophages [114]. Transcriptome analyses indicate enhanced resilience of endosymbiont-bearing *Rhizopus* species in stress situations, such as phagocytosis by macrophages [141]. The bacterial endosymbionts induced various transcriptomic responses only when the host fungus was subjected to external stress, emphasizing the supportive role during immune system evasion [141].

Metabolites from endofungal bacteria symbiotically associated with a *R. microsporus* strain may have also contributed to the worsening of a local infection in a human [108]. *R. microsporus* was transferred through a spider bite into the small finger of a woman in Australia [108] (Fig. 4C). The transferred fungus caused a severe infection at the bite site on the hand, resulting in tissue necrosis [74]. After progressive inflammation at the encounter site and a general worsening of the health condition of the woman, an amputation of the little finger and later the whole hand was inevitable [74]. The severity of the necrosis may have been exacerbated by the cytotoxin necroxime. Necroxime is a cytotoxic and antiproliferative benzolactone with activity at concentrations as low as 0.6 μM [108]. It is structurally related to other benzolactones that inhibit V-ATPases [142]. Again, the toxin is not produced by the fungus but by endobacteria, a unique branch of *Mycetohabitans* sp. (strain HKI-404).

The previously mentioned influences of endofungal bacteria during systemic or local infections with *R. microsporus* in humans suggest to more regularly check for the presence of bacterial symbionts that might influence the progression of a fungal infection. Such a procedure might allow more individually customized treatment options leading to better outcomes for patients.

### Endofungal bacteria provide defence against predators

Benzolactones related to necroximes have also been isolated from cultures of a specific *P. verticillata* strain [143]. The compounds, originally named CJ-12950 and CJ-13357, were known for a long time and believed to be of fungal origin, similar to rhizoxins and rhizonins [18, 109, 143]. However, biosynthesis of the metabolites has been associated in later studies with a so far unknown endofungal bacterium, *Ca.* Mycoavidus necroximicus [92]. A corresponding biosynthetic gene cluster, nearly identical to the gene cluster known to encode the biosynthetic machinery for necroxime production from the *Mycetohabitans* endosymbiont of *R. microsporus*, could be identified in *Ca.* Mycoavidus necroximicus [92, 108]. These necroxim toxins are potent anthelmintica, acting as a chemical shield to protect the fungal host from nematodal predators (Fig. 4D) [92]. In addition, another endobacteria-derived metabolite, symbiosin, synergistically enhances the nematocidal effect of necroxime, thereby amplifying the host protection [112].

Anthelmintic protection is not unique for *P. verticillata.* Although the Mortierellomycotina fungus *Mortierella alpina* has no endosymbiotic bacteria (anymore), genes encoding a bacterial-like biosynthetic machinery that synthesizes the anthelminticum malpinin, can be identified in the fungal genome [144, 145]. It is suspected that the malpinin-gene cluster is the remain of an ancient, now obsolete, endofungal bacterium, which could have been responsible for chemical protection of the fungus against predators [146].

The strategy to facilitate protection against predators with help of metabolites produced by endofungal bacteria is convenient for fungi. Environmental *R. microsporus* strains without plant-pathogenic traits also produce rhizoxin-congeners, leading to the assumption that the aforementioned rhizoxins of the *Rhizopus*-*Mycetohabitans* symbiosis may have dual functions [93]. Besides phytopathogenic activities, anthelmintic and amoebicidal bioassays suggest an additional protective function of these metabolites against fungivorous nematodes and amoebae (Fig. 4D) [93]. Rhizoxins efficiently protect fungal spores from amoebal phagocytosis and inhibit fungivorous nematodes [93]. This involvement in the fungal protection against protozoan and metazoan micropredators may have originally developed to provide protection against fungal predators and only later facilitated the emergence of plant pathogenicity [93]. The interactions between Mucoromycota and their bacterial endosymbionts are an ancient phenomenon dating back as far as 400 million years [147]. Flowering land plants like rice developed far later (134 million years ago) than protozoan and metazoan predators such as amoebae and nematodes (400 million years ago) [148, 149]. Thus, the establishment of a mutualistic interaction between *Rhizopus* and rhizoxin-producing *Mycetohabitans* [58] may have allowed the fungal host to evade predator attack thereby gaining an evolutionary advantage over endosymbiont-free or rhizoxin-negative symbiotic fungi.

In addition to the *Mycetohabitans*-mediated protection of *R. microsporus* against amoeba, there are other endosymbionts of *R. microsporus* that confer protection against these protozoan predators. The bacterial endosymbiont *R. pickettii,* detected in a clinical isolate of *R. microsporus,* also protects the spores of its fungal host from amoebal ingestion [114]. *R. pickettii* secretes an unknown factor responsible for killing amoebae in the environment [114]. It is proposed that such interactions with amoebae provide a training ground for fungi to evade the human immune system, i.e. clearance by macrophages [150–153]. Thus, such environmentally evolved defensive mechanisms may play an additional role in tripartite interactions of endosymbiont-harbouring fungi with humans.

In general, the pathogenicity mechanisms that have evolved in *R. microsporus* and other clinically important fungi during interactions with amoebae are equally effective during encounters with macrophages of the vertebrate immune system, including humans. Amoebae and macrophages have a similar morphology, and their phagocytic processes are analogous. Mechanisms that evolved to be effective against amoeba in the environment may also be effective against macrophages, explaining the persistence and even the evolution of these traits [150–153].

## Conclusions

As research into bacterial-fungal endosymbiosis continues to grow, we are beginning to understand how these interactions affect not only the individual symbiosis partners but also their relationships with the environment and higher-order interactions. This review shows that bacterial-fungal endosymbiosis is a dynamic relationship, characterized by intricate molecular interactions that can shift along a spectrum from mutualism to parasitism. This dynamic nature is reflected in the fact that endofungal bacteria often exhibit parasitic traits, such as their dependence on host-derived resources, while at the same time contributing beneficially by enhancing fungal growth, stress tolerance, and metabolite production. Ultimately, whether bacterial-fungal endosymbiosis is beneficial or detrimental likely depends on the influence of endofungal bacteria on the fungal host's interactions with the environment and other organisms. For example, endofungal bacteria contribute to the beneficial effects of fungi on plant health and fitness, protect their fungal host from fungivorous predators, and enhance fungal virulence against plants, animals, and humans.

Although tripartite interactions that promote plant growth and health have been studied for decades, our knowledge of the impact of bacterial-fungal endosymbiosis on other organisms is still in its infancy. Whereas a limited number of studies have highlighted the importance of bacterially produced molecules on animals and humans, the molecular details underlying these tripartite interactions remain largely unknown. Moreover, these studies are primarily laboratory-based and focus on a single organism interacting with a bacterial-fungal pair. This raises the question of whether endofungal bacteria exert the same influence on animals, humans, and plants in their native environments. Recent insights from bacterial systems suggest that the structure and stability of microbial communities can be shaped by higher-order competitive dynamics, including both transitive hierarchies and intransitive loops, where no single partner consistently outcompetes the others. These complex interaction patterns, which go beyond simple pairwise relationships, may play a key role in maintaining microbial diversity and shaping the ecological outcomes of endosymbiotic associations [154]. In order to better understand the role of endofungal bacteria in microbial communities, future research should aim to elucidate these interactions at a mechanistic level and incorporate field-based studies. An integrated understanding of endofungal bacteria not only advances our knowledge of microbial ecology and ecosystem functioning, but also has significant implications for medicine, agriculture, food processing, and biotechnology.

Understanding the role of endofungal bacteria in ecosystem functioning and fungal virulence opens promising avenues for applications in medicine and agriculture. In a therapeutic context, fungal infections can be addressed by developing strategies that specifically target pathogenic endofungal bacteria, rather than directly harming the fungal host. This approach could provide an effective alternative or complementary approach for the treatment of fungal pathogens, which often exhibit inherent resistance to antifungal drugs. In agriculture, genetic manipulation of host-protective endofungal bacteria may improve plant and soil health, reducing reliance on chemical pesticides while increasing crop resilience and productivity. These advances underscore the potential of endofungal bacteria to drive both sustainable farming practices and innovative medical therapies.

## Data Availability

Data sharing is not applicable to this article as no datasets were generated or analysed during the current study.

## References

[1] Bahram M, Netherway T. Fungi as mediators linking organisms and ecosystems. *FEMS Microbiol Rev* 2022;46:fuab058. 10.1093/femsre/fuab058 34919672 PMC8892540

[2] Netherway T, Bengtsson J, Krab EJ. et al. Biotic interactions with mycorrhizal systems as extended nutrient acquisition strategies shaping forest soil communities and functions. *Basic Appl Ecol* 2021;50:25–42. 10.1016/j.baae.2020.10.002

[3] Tedersoo L, Bahram M, Zobel M. How mycorrhizal associations drive plant population and community biology. *Science* 2020;367:eaba1223. 10.1126/science.aba1223 32079744

[4] Fisher MC, Henk DA, Briggs CJ. et al. Emerging fungal threats to animal, plant and ecosystem health. *Nature* 2012;484:186–94. 10.1038/nature10947 22498624 PMC3821985

[5] Steffan BN, Venkatesh N, Keller NP. Let's get physical: bacterial-fungal interactions and their consequences in agriculture and health. *J Fungi* 2020;6:243. 10.3390/jof6040243 PMC7712096 33114069

[6] Pawlowska TE . Symbioses between fungi and bacteria: from mechanisms to impacts on biodiversity. *Curr Opin Microbiol* 2024;80:102496. 10.1016/j.mib.2024.102496 38875733 PMC11323152

[7] Duan S, Feng G, Limpens E. et al. Cross-kingdom nutrient exchange in the plant–arbuscular mycorrhizal fungus–bacterium continuum. *Nat Rev Microbiol* 2024;12:773–90. 10.1038/s41579-024-01073-7 39014094

[8] Alabid I, Glaeser SP, Kogel KH. Endofungal bacteria increase fitness of their host fungi and impact their association with crop plants. *Curr Issues Mol Biol* 2019;30:59–74. 10.21775/cimb.030.059 30070651

[9] Arora P, Riyaz-Ul-Hassan S. Endohyphal bacteria; the prokaryotic modulators of host fungal biology. *Fungal Biol Rev* 2019;33:72–81. 10.1016/j.fbr.2018.08.003

[10] Scherlach K, Graupner K, Hertweck C. Molecular bacteria-fungi interactions: effects on environment, food, and medicine. *Ann Rev Microbiol* 2013;67:375–97. 10.1146/annurev-micro-092412-155702 23808337

[11] Scherlach K, Hertweck C. Chemical mediators at the bacterial-fungal interface. *Ann Rev Microbiol* 2020;74:267–90. 10.1146/annurev-micro-012420-081224 32660387

[12] Lackner G, Hertweck C. Impact of endofungal bacteria on infection biology, food safety, and drug development. *PLoS Pathog* 2011;7:4. 10.1371/journal.ppat.1002096 PMC3128126 21738468

[13] Deveau A, Bonito G, Uehling J. et al. Bacterial-fungal interactions: ecology, mechanisms and challenges. *FEMS Microbiol Rev* 2018;42:335–52. 10.1093/femsre/fuy008 29471481

[14] Mosse B . Honey-coloured, sessile Endogone spores: II. Changes in fine structure during spore development. *Arch Microbiol* 1970;74:129–45. 10.1007/BF00446901

[15] Macdonald RM, Chandler MR, Mosse B. The occurrence of bacterium-like organelles in vesicular-arbuscular mycorrhizal fungi. *The New Phytol* 1982;90:659–63. 10.1111/j.1469-8137.1982.tb03275.x

[16] Bonfante-Fasolo P, Scannerini S. The ultrastructure of the zygospore in *Endogone flammicorona* Trappe & Gerdemann. *Mycopathologia* 1976;59:117–23. 10.1007/BF00493564

[17] Scannerini S, Bonfante P. In: Margulis L, Fester R. (eds.), Symbiosis as a Source of Evolutionary Innovation: Speciation and Morphogenesis. Cambridge, MA, USA: MIT Press, 1991, 273–87. 11538111

[18] Partida-Martinez LP, Hertweck C. Pathogenic fungus harbours endosymbiotic bacteria for toxin production. *Nature* 2005;437:884–8. 10.1038/nature03997 16208371

[19] Scherlach K, Busch B, Lackner G. et al. Symbiotic cooperation in the biosynthesis of a phytotoxin. *Angew Chem Int Ed* 2012;51:9615–8. 10.1002/anie.201204540 22915379

[20] Partida-Martinez LP, Monajembashi S, Greulich KO. et al. Endosymbiont-dependent host reproduction maintains bacterial-fungal mutualism. *Curr Biol* 2007;17:773–7. 10.1016/j.cub.2007.03.039 17412585

[21] Partida-Martinez LP, Hertweck C. A gene cluster encoding rhizoxin biosynthesis in *Burkholderia rhizoxina*, the bacterial endosymbiont of the fungus *Rhizopus microsporus*. *ChemBioChem* 2007;8:41–5. 10.1002/cbic.200600393 17154220

[22] Lumini E, Bianciotto V, Jargeat P. et al. Presymbiotic growth and sporal morphology are affected in the arbuscular mycorrhizal fungus *Gigaspora margarita* cured of its endobacteria. *Cell Microbiol* 2007;9:1716–29. 10.1111/j.1462-5822.2007.00907.x 17331157

[23] Hoffman MT, Arnold AE. Diverse bacteria inhabit living hyphae of phylogenetically diverse fungal endophytes. *Appl Environ Microbiol* 2010;76:4063–75. 10.1128/AEM.02928-09 20435775 PMC2893488

[24] Arendt KR, Hockett KL, Araldi-Brondolo SJ. et al. Isolation of endohyphal bacteria from foliar Ascomycota and *in vitro* establishment of their symbiotic associations. *Appl Environ Microbiol* 2016;82:2943–9. 10.1128/AEM.00452-16 26969692 PMC4959084

[25] Bertaux J, Schmid M, Prevost-Boure NC. et al. *In situ* identification of intracellular bacteria related to *Paenibacillus* spp. in the mycelium of the ectomycorrhizal fungus *Laccaria bicolor* S238N. *Appl Environ Microbiol* 2003;69:4243–8. 10.1128/AEM.69.7.4243-4248.2003 12839806 PMC165139

[26] Bertaux J, Schmid M, Hutzler P. et al. Occurrence and distribution of endobacteria in the plant-associated mycelium of the ectomycorrhizal fungus *Laccaria bicolor* S238N. *Environ Microbiol* 2005;7:1786–95. 10.1111/j.1462-2920.2005.00867.x 16232293

[27] Sharma M, Schmid M, Rothballer M. et al. Detection and identification of bacteria intimately associated with fungi of the order *Sebacinales*. *Cell Microbiol* 2008;10:2235–46. 10.1111/j.1462-5822.2008.01202.x 18637023

[28] Ruiz-Herrera J, León-Ramírez C, Vera-Nuñez A. et al. A novel intracellular nitrogen-fixing symbiosis made by *Ustilago maydis* and *Bacillus* spp. *New Phytol* 2015;207:769–77. 10.1111/nph.13359 25754368

[29] Liang P, Jiang J, Sun Z. et al. *Klebsiella michiganensis*: a nitrogen-fixing endohyphal bacterium from *Ustilago maydis*. *AMB Express* 2023;13:146. 10.1186/s13568-023-01618-8 38112810 PMC10730499

[30] Pérez-Rodríguez F, González-Prieto JM, Vera-Núñez JA. et al. Wide distribution of the *Ustilago maydis*-bacterium endosymbiosis in naturally infected maize plants. *Plant Signal Behav* 2021;16:1855016. 10.1080/15592324.2020.1855016 33356903 PMC7849723

[31] Uehling JK, Salvioli A, Amses KR. et al. Bacterial endosymbionts of Mucoromycota fungi: Diversity and function of their interactions in Evolution of Fungi and Fungal-like Organisms. Pöggeler S, James T (eds.), Cham: Springer International Publishing, 2023, 177–205. 10.1007/978-3-031-29199-9_8

[32] Bonfante P, Desirò A. Who lives in a fungus? The diversity, origins and functions of fungal endobacteria living in Mucoromycota. *ISME J.* 2017;11:1727–35. 10.1038/ismej.2017.21 28387771 PMC5520026

[33] Okrasińska A, Bokus A, Duk K. et al. New endohyphal relationships between Mucoromycota and *Burkholderiaceae* representatives. *Appl Environ Microbiol* 2021;87:e02707–20. 10.1128/AEM.02707-20 33483310 PMC8091615

[34] Bonfante P, Venice F. Mucoromycota: going to the roots of plant-interacting fungi. *Front Microbiol* 2020;34:100–13. 10.1016/j.fbr.2019.12.003

[35] Uehling J, Gryganskyi A, Hameed K. et al. Comparative genomics of *Mortierella elongata* and its bacterial endosymbiont *Mycoavidus cysteinexigens*. *Environ Microbiol* 2017;19:2964–83. 10.1111/1462-2920.13669 28076891

[36] Ohshima S, Sato Y, Fujimura R. et al. *Mycoavidus cysteinexigens* gen. Nov., sp. nov., an endohyphal bacterium isolated from a soil isolate of the fungus *Mortierella elongata*. *Int J Syst Evol Microbiol* 2016;66:2052–7. 10.1099/ijsem.0.000990 26920389

[37] Wernegreen JJ . Endosymbiosis. *Curr Biol* 2012;22:R555–61. 10.1016/j.cub.2012.06.010 22835786

[38] Keeling PJ . The endosymbiotic origin, diversification and fate of plastids. *Philos Trans R Soc Lond Ser B Biol Sci* 2010;365:729–48. 10.1098/rstb.2009.0103 20124341 PMC2817223

[39] Nowack ECM, Melkonian M. Endosymbiotic associations within protists. *Philos Trans R Soc Lond Ser B Biol Sci* 2010;365:699–712. 10.1098/rstb.2009.0188 20124339 PMC2817226

[40] Moore RB, Oborník M, Janouskovec J. et al. A photosynthetic alveolate closely related to apicomplexan parasites. *Nature* 2008;451:959–63. 10.1038/nature06635 18288187

[41] Costa TRD, Felisberto-Rodrigues C, Meir A. et al. Secretion systems in gram-negative bacteria: structural and mechanistic insights. *Nat Rev Microbiol* 2015;13:343–59. 10.1038/nrmicro3456 25978706

[42] Zhang P, Huguet-Tapia J, Peng Z. et al. Genome analysis and hyphal movement characterization of the hitchhiker endohyphal *Enterobacter* sp. from *Rhizoctonia solani*. *Appl Environ Microbiol* 2024;90:e02245–23. 10.1128/aem.02245-23 38319098 PMC10952491

[43] Liu X-L, Zhao H, Wang Y-X. et al. Detecting and characterizing new endofungal bacteria in new hosts: *Pandoraea sputorum* and *Mycetohabitans endofungorum* in *Rhizopus arrhizus*. *Front Microbiol* 2024;15:1346252. 10.3389/fmicb.2024.1346252 38486702 PMC10939042

[44] Baltrus DA, Dougherty K, Arendt KR. et al. Absence of genome reduction in diverse, facultative endohyphal bacteria. *Microb Genom* 2017;3:e000101. 10.1099/mgen.0.000101 28348879 PMC5361626

[45] Lastovetsky OA, Krasnovsky LD, Xiaotian Q. et al. Molecular dialogues between early divergent fungi and bacteria in an antagonism versus a mutualism. *mBio* 2020;11:e02088–20. 10.1128/mBio.02088-20 32900811 PMC7482071

[46] Lackner G, Moebius N, Hertweck C. Endofungal bacterium controls its host by an hrp type III secretion system. *ISME J* 2011;5:252–61. 10.1038/ismej.2010.126 20720578 PMC3105691

[47] Niehs SP, Scherlach K, Hertweck C. Genomics-driven discovery of a linear lipopeptide promoting host colonization by endofungal bacteria. *Org Biomol Chem* 2018;16:8345–52. 10.1039/C8OB01515E 30209475

[48] Spraker JE, Sanchez LM, Lowe TM. et al. *Ralstonia solanacearum* lipopeptide induces chlamydospore development in fungi and facilitates bacterial entry into fungal tissues. *ISME J.* 2016;10:2317–30. 10.1038/ismej.2016.32 26943626 PMC4989320

[49] Venkatesh N, Greco C, Drott MT. et al. Bacterial hitchhikers derive benefits from fungal housing. *Curr Biol* 2022;32:1523–33. 10.1016/j.cub.2022.02.017 35235767 PMC9009100

[50] Hazarika DJ, Gautom T, Parveen A. et al. Mechanism of interaction of an endofungal bacterium *Serratia marcescens* D1 with its host and non-host fungi. *PLoS One* 2020;15:e0224051. 10.1371/journal.pone.0224051 32320394 PMC7176118

[51] Sharmin D, Guo Y, Nishizawa T. et al. Comparative genomic insights into endofungal lifestyles of two bacterial endosymbionts, *Mycoavidus cysteinexigens* and *Burkholderia rhizoxinica*. *Microbes Environ* 2018;33:66–76. 10.1264/jsme2.ME17138 29540638 PMC5877345

[52] Moebius N, Uzum Z, Dijksterhuis J. et al. Active invasion of bacteria into living fungal cells. *eLife* 2014;3:e03007. 10.7554/eLife.03007 25182414 PMC4166002

[53] Shaffer JP, Carter ME, Spraker JE. et al. Transcriptional profiles of a foliar fungal endophyte (*Pestalotiopsis*, Ascomycota) and its bacterial symbiont (*Luteibacter*, Gammaproteobacteria) reveal sulfur exchange and growth regulation during early phases of symbiotic interaction. *mSystems* 2022;7:e0009122. 10.1128/msystems.00091-22 35293790 PMC9040847

[54] Salvioli A, Ghignone S, Novero M. et al. Symbiosis with an endobacterium increases the fitness of a mycorrhizal fungus, raising its bioenergetic potential. *ISME J.* 2016;10:130–44. 10.1038/ismej.2015.91 26046255 PMC4681866

[55] Ghignone S, Salvioli A, Anca I. et al. The genome of the obligate endobacterium of an AM fungus reveals an interphylum network of nutritional interactions. *ISME J.* 2012;6:136–45. 10.1038/ismej.2011.110 21866182 PMC3246228

[56] Giger GH, Ernst C, Richter I. et al. Inducing novel endosymbioses by implanting bacteria in fungi. *Nature* 2024;635:415–22. 10.1038/s41586-024-08010-x 39358514 PMC11560845

[57] Jwa NS, Hwang BK. Convergent evolution of pathogen effectors toward reactive oxygen species signaling networks in plants. *Front Plant Sci* 2017;8:01687. 10.3389/fpls.2017.01687 PMC5627460 29033963

[58] Lastovetsky OA, Gaspar ML, Mondo SJ. et al. Lipid metabolic changes in an early divergent fungus govern the establishment of a mutualistic symbiosis with endobacteria. *Proc Natl Acad Sci USA* 2016;113:15102–7. 10.1073/pnas.1615148113 27956601 PMC5206550

[59] Leone MR, Lackner G, Silipo A. et al. An unusual galactofuranose lipopolysaccharide that ensures the intracellular survival of toxin-producing bacteria in their fungal host. *Angew Chem Int Ed* 2010;49:7476–80. 10.1002/anie.201003301 20718018

[60] Richter I, Wein P, Uzum Z. et al. Transcription activator-like effector protects bacterial endosymbionts from entrapment within fungal hyphae. *Curr Biol* 2023;33:2646–56. 10.1016/j.cub.2023.05.028 37301202 PMC10337650

[61] Richter I, Uzum Z, Wein P. et al. Transcription activator-like effectors from endosymbiotic bacteria control the reproduction of their fungal host. *mBio* 2023;14:e0182423. 10.1128/mbio.01824-23 37971247 PMC10746252

[62] Pérez-Brocal V, Latorre A, Moya A. In: Between Pathogenicity and Commensalism. Dobrindt U, Hacker JH, Svanborg C (eds.) Berlin, Heidelberg, Germnay: Springer International Publishing, 2013, 215–43. 10.1007/82_2011_190

[63] Lackner G, Moebius N, Partida-Martinez L. et al. Complete genome sequence of *Burkholderia rhizoxinica*, an endosymbiont of *Rhizopus microsporus*. *J Bacteriol* 2011;193:783–4. 10.1128/JB.01318-10 21131495 PMC3021220

[64] Niehs SP, Scherlach K, Dose B. et al. A highly conserved gene locus in endofungal bacteria codes for the biosynthesis of symbiosis-specific cyclopeptides. *PNAS Nexus* 2022;1:pgac152. 10.1093/pnasnexus/pgac152 36714835 PMC9802438

[65] Scherlach K, Partida-Martinez LP, Dahse HM. et al. Antimitotic rhizoxin derivatives from a cultured bacterial endosymbiont of the rice pathogenic fungus *Rhizopus microsporus*. *J Am Chem Soc* 2006;128:11529–36. 10.1021/ja062953o 16939276

[66] Schmitt I, Partida-Martinez LP, Winkler R. et al. Evolution of host resistance in a toxin-producing bacterial-fungal alliance. *ISME J* 2008;2:632–41. 10.1038/ismej.2008.19 18309361

[67] Chávez-González JD, Flores-Núñez VM, Merino-Espinoza IU. et al. Desert plants, arbuscular mycorrhizal fungi and associated bacteria: exploring the diversity and role of symbiosis under drought. *Environ Microbiol Rep* 2024;16:e13300. 10.1111/1758-2229.13300 38979873 PMC11231939

[68] Bianciotto V, Genre A, Jargeat P. et al. Vertical transmission of endobacteria in the arbuscular mycorrhizal fungus *Gigaspora margarita* through generation of vegetative spores. *Appl Environ Microbiol* 2004;70:3600–8. 10.1128/AEM.70.6.3600-3608.2004 15184163 PMC427789

[69] Lastovetsky OA, Caruso T, Brennan FP. et al. Spores of arbuscular mycorrhizal fungi host surprisingly diverse communities of endobacteria. *New Phytol* 2024;242:1785–97. 10.1111/nph.19605 38403930

[70] Takashima Y, Degawa Y, Nishizawa T. et al. Aposymbiosis of a *Burkholderiaceae*-related endobacterium impacts on sexual reproduction of its fungal host. *Microbes Environ* 2020;35:ME19167. 10.1264/jsme2.ME19167 32295978 PMC7308579

[71] Mondo SJ, Lastovetsky OA, Gaspar ML. et al. Bacterial endosymbionts influence host sexuality and reveal reproductive genes of early divergent fungi. *Nat Commun* 2017;8:1843–3. 10.1038/s41467-017-02052-8 29184190 PMC5705715

[72] Wyatt TT, Wösten HA, Dijksterhuis J. Fungal spores for dispersion in space and time. *Adv Appl Microbiol* 2013;85:43–91. 10.1016/B978-0-12-407672-3.00002-2 23942148

[73] Lackner G, Mobius N, Scherlach K. et al. Global distribution and evolution of a toxinogenic *Burkholderia*-*Rhizopus* symbiosis. *Appl Environ Microbiol* 2009;75:2982–6. 10.1128/AEM.01765-08 19286793 PMC2681710

[74] Kerr CP, Turner H, Davidson A. et al. Zygomycosis requiring amputation of the hand: an isolated case in a patient receiving haemodialysis. *Med J Aust* 1988;148:258–9. 10.5694/j.1326-5377.1988.tb99438.x 3343958

[75] Cabrera-Rangel JF, Mendoza-Servín JV, Córdova-López G. et al. Symbiotic and toxinogenic *Rhizopus* spp. isolated from soils of different papaya producing regions in Mexico. *Front Fungal Biol* 2022;3:893700. 10.3389/ffunb.2022.893700 37746220 PMC10512248

[76] Niehs SP, Dose B, Scherlach K. et al. Genomics-driven discovery of a symbiont-specific cyclopeptide from bacteria residing in the rice seedling blight fungus. *ChemBioChem* 2018;19:2167–72. 10.1002/cbic.201800400 30113119

[77] Carpenter SCD, Bogdanove AJ, Abbot B. et al. Prevalence and diversity of TAL effector-like proteins in fungal endosymbiotic *Mycetohabitans* spp. *Microb Genom* 2024;10:001261. 10.1099/mgen.0.001261 38860878 PMC11261895

[78] Naumann M, Schüssler A, Bonfante P. The obligate endobacteria of arbuscular mycorrhizal fungi are ancient heritable components related to the Mollicutes. *ISME J.* 2010;4:862–71. 10.1038/ismej.2010.21 20237515

[79] Lastovetsky OA, Ahn E, Mondo SJ. et al. Distribution and population structure of endobacteria in arbuscular mycorrhizal fungi at North Atlantic dunes. *ISME J.* 2018;12:3001–13. 10.1038/s41396-018-0246-2 30097664 PMC6246606

[80] Guo H, Glaeser SP, Alabid I. et al. The abundance of endofungal bacterium *rhizobium radiobacter* (syn. *Agrobacterium tumefaciens*) increases in its fungal host *Piriformospora indica* during the tripartite *Sebacinalean* symbiosis with higher plants. *Front Microbiol* 2017;8:00629. 10.3389/fmicb.2017.00629 PMC5390018 28450855

[81] Obasa K, Adesemoye A, Obasa R. et al. Endohyphal bacteria associated with virulence, increased expression of fumonisin biosynthetic genes, and production of fumonisin and macroconidia in *Fusarium fujikuroi* W343. *Plant Pathol J* 2020;69:87–100. 10.1111/ppa.13107

[82] Espino-Vázquez AN, Bermúdez-Barrientos JR, Cabrera-Rangel JF. et al. Narnaviruses: novel players in fungal-bacterial symbioses. *ISME J.* 2020;14:1743–54. 10.1038/s41396-020-0638-y 32269378 PMC7305303

[83] Yamamura N . Vertical transmission and evolution of mutualism from parasitism. *Theor Popul Biol* 1993;44:95–109. 10.1006/tpbi.1993.1020

[84] Naito M, Morton JB, Pawlowska TE. Minimal genomes of mycoplasma-related endobacteria are plastic and contain host-derived genes for sustained life within Glomeromycota. *Proc Natl Acad Sci USA* 2015;112:7791–6. 10.1073/pnas.1501676112 25964324 PMC4485128

[85] Toomer KH, Chen X, Naito M. et al. Molecular evolution patterns reveal life history features of *mycoplasma*-related endobacteria associated with arbuscular mycorrhizal fungi. *Mol Ecol* 2015;24:3485–500. 10.1111/mec.13250 26011293

[86] Koch AM, Kuhn G, Fontanillas P. et al. High genetic variability and low local diversity in a population of arbuscular mycorrhizal fungi. *Proc Natl Acad Sci USA* 2004;101:2369–74. 10.1073/pnas.0306441101 14983016 PMC356957

[87] Savary R, Dupuis C, Masclaux FG. et al. Genetic variation and evolutionary history of a mycorrhizal fungus regulate the currency of exchange in symbiosis with the food security crop cassava. *ISME J.* 2020;14:1333–44. 10.1038/s41396-020-0606-6 32066875 PMC7242447

[88] Savary R, Masclaux FG, Sanders IR. The model arbuscular mycorrhizal fungus *Rhizophagus irregularis* harbours endosymbiotic bacteria with a highly reduce genome. *bioRxiv* 2021. 10.1101/2021.09.13.460061

[89] Frank SA . Host–symbiont conflict over the mixing of symbiotic lineages. *Proc R Soc Lond B Biol Sci* 1996;263:339–44. 10.1098/rspb.1996.0052 8920255

[90] Moran NA, McCutcheon JP, Nakabachi A. Genomics and evolution of heritable bacterial symbionts. *Annu Rev Genet* 2008;42:165–90. 10.1146/annurev.genet.41.110306.130119 18983256

[91] Bronstein JL . Conditional outcomes in mutualistic interactions. *Trends Ecol Evol* 1994;9:214–7. 10.1016/0169-5347(94)90246-1 21236825

[92] Büttner H, Niehs SP, Vandelannoote K. et al. Bacterial endosymbionts protect beneficial soil fungus from nematode attack. *Proc Natl Acad Sci USA* 2021;118:e2110669118. 10.1073/pnas.2110669118 34504005 PMC8449335

[93] Richter I, Radosa S, Cseresnyés Z. et al. Toxin-producing endosymbionts shield pathogenic fungus against micropredators. *mBio* 2022;13:e01440–22. 10.1128/mbio.01440-22 36005392 PMC9600703

[94] Amses K, Amses K, Desiró A. et al. Convergent reductive evolution and host adaptation in *Mycoavidus* bacterial endosymbionts of *Mortierellaceae* fungi. *Fungal Genet Biol* 2023;169:103838. 10.1016/j.fgb.2023.103838 37716699

[95] Valadez-Cano C, Olivares-Hernández R, Espino-Vázquez AN. et al. Genome-scale model of *Rhizopus microsporus*: metabolic integration of a fungal holobiont with its bacterial and viral endosymbionts. *Environ Microbiol* 2024;26:e16551. 10.1111/1462-2920.16551 38072824

[96] Audia JP, Winkler HH. Study of the five *Rickettsia prowazekii* proteins annotated as ATP/ADP translocases (Tlc): only Tlc1 transports ATP/ADP, while Tlc4 and Tlc5 transport other ribonucleotides. *J Bacteriol* 2006;188:6261–8. 10.1128/JB.00371-06 16923893 PMC1595366

[97] Meidanis J, Braga MD, Verjovski-Almeida S. Whole-genome analysis of transporters in the plant pathogen *Xylella fastidiosa*. *Microbiol Mol Biol Rev* 2002;66:272–99. 10.1128/MMBR.66.2.272-299.2002 12040127 PMC120790

[98] Mares-Rodriguez F. De J, Aréchiga-Carvajal ET, Ruiz-Herrera J. et al. A new bacterial endosymbiotic relationship in *Kluyveromyces marxianus* isolated from the mezcal fermentation process. *Process Biochem* 2023;131:133–43. 10.1016/j.procbio.2023.06.008

[99] Carter ME, Carpenter SCD, Dubrow ZE. et al. A TAL effector-like protein of an endofungal bacterium increases the stress tolerance and alters the transcriptome of the host. *Proc Natl Acad Sci USA* 2020;117:17122–9. 10.1073/pnas.2003857117 32632014 PMC7382252

[100] Niehs SP, Dose B, Scherlach K. et al. Genome mining reveals endopyrroles from a nonribosomal peptide assembly line triggered in fungal-bacterial symbiosis. *ACS Chem Biol* 2019;14:1811–8. 10.1021/acschembio.9b00406 31283172

[101] Lupini S, Peña-Bahamonde J, Bonito G. et al. Effect of endosymbiotic bacteria on fungal resistance toward heavy metals. *Front Microbiol* 2022;13:822541. 10.3389/fmicb.2022.822541 35369521 PMC8965456

[102] Shaffer JP, U'Ren JM, Gallery RE. et al. An endohyphal bacterium (*Chitinophaga*, Bacteroidetes) alters carbon source use by *Fusarium keratoplasticum* (*F. Solani* species complex, Nectriaceae). *Front Microbiol* 2017;8:00350. 10.3389/fmicb.2017.00350 PMC5361657 28382021

[103] Desirò A, Hao Z, Liber JA. et al. Mycoplasma-related endobacteria within Mortierellomycotina fungi: diversity, distribution and functional insights into their lifestyle. *ISME J.* 2018;12:1743–57. 10.1038/s41396-018-0053-9 29476142 PMC6018737

[104] Moran NA, Plague GR, Sandström JP. et al. A genomic perspective on nutrient provisioning by bacterial symbionts of insects. *Proc Natl Acad Sci USA* 2003;100:14543–8. 10.1073/pnas.2135345100 14527994 PMC304116

[105] Braga D, Last D, Hasan M. et al. Metabolic pathway rerouting in *Paraburkholderia rhizoxinica* evolved long-overlooked derivatives of coenzyme F_420_. *ACS Chem Biol* 2019;14:2088–94. 10.1021/acschembio.9b00605 31469543

[106] Richter I, Hasan M, Kramer JW. et al. Deazaflavin metabolite produced by endosymbiotic bacteria controls fungal host reproduction. *ISME J.* 2024;18:wrae074. 10.1093/ismejo/wrae074 38691425 PMC11104420

[107] Isabelle D, Simpson DR, Daniels L. Large-scale production of coenzyme F_420_-5,6 by using *mycobacterium smegmatis*. *Appl Environ Microbiol* 2002;68:5750–5. 10.1128/AEM.68.11.5750-5755.2002 12406775 PMC129890

[108] Niehs SP, Dose B, Richter S. et al. Mining symbionts of a spider-transmitted fungus illuminates uncharted biosynthetic pathways to cytotoxic benzolactones. *Angew Chem Int Ed* 2020;59:7766–71. 10.1002/anie.201916007 PMC7318616 32040253

[109] Partida-Martinez LP, de Looss CF, Ishida K. et al. Rhizonin, the first mycotoxin isolated from the Zygomycota, is not a fungal metabolite but is produced by bacterial endosymbionts. *Appl Environ Microbiol* 2007;73:793–7. 10.1128/AEM.01784-06 17122400 PMC1800748

[110] Wang X, Zhou H, Chen H. et al. Discovery of recombinases enables genome mining of cryptic biosynthetic gene clusters in Burkholderiales species. *Proc Natl Acad Sci USA* 2018;115:E4255–63. 10.1073/pnas.1720941115 29666226 PMC5939090

[111] Bratovanov EV, Ishida K, Heinze B. et al. Genome mining and heterologous expression reveal two distinct families of lasso peptides highly conserved in endofungal bacteria. *ACS Chem Biol* 2020;15:1169–76. 10.1021/acschembio.9b00805 31800204

[112] Büttner H, Pidot SJ, Scherlach K. et al. Endofungal bacteria boost anthelminthic host protection with the biosurfactant symbiosin. *Chem Sci* 2022;14:103–12. 10.1039/D2SC04167G 36605741 PMC9769094

[113] Hoffman MT, Gunatilaka MK, Wijeratne K. et al. Endohyphal bacterium enhances production of indole-3-acetic acid by a foliar fungal endophyte. *PLoS One* 2013;8:e73132. 10.1371/journal.pone.0073132 24086270 PMC3782478

[114] Itabangi H, Sephton-Clark P, Tamayo DP. et al. A bacterial endosymbiont of the fungus *Rhizopus microsporus* drives phagocyte evasion and opportunistic virulence. *Curr Biol* 2022;32:1115–30. 10.1016/j.cub.2022.01.028 35134329 PMC8926845

[115] Parniske M . Arbuscular mycorrhiza: the mother of plant root endosymbioses. *Nat Rev Microbiol* 2008;6:763–75. 10.1038/nrmicro1987 18794914

[116] Shi J, Wang X, Wang E. Mycorrhizal symbiosis in plant growth and stress adaptation: from genes to ecosystems. *Annu Rev Plant Biol* 2023;74:569–607. 10.1146/annurev-arplant-061722-090342 36854473

[117] Ciach MA, Pawłowska J, Górecki P. et al. The interkingdom horizontal gene transfer in 44 early diverging fungi boosted their metabolic, adaptive, and immune capabilities. *Evol Lett* 2024;8:526–38. 10.1093/evlett/qrae009 39100235 PMC11291939

[118] Sangwan S, Prasanna R. Mycorrhizae helper bacteria: unlocking their potential as bioenhancers of plant–arbuscular mycorrhizal fungal associations. *Microb Ecol* 2022;84:1–10. 10.1007/s00248-021-01831-7 34417849

[119] Araldi-Brondolo SJ, Spraker J, Shaffer JP. et al. Bacterial endosymbionts: master modulators of fungal phenotypes. *Microbiol Spectr* 2017;5:056. 10.1128/microbiolspec.funk-0056-2016 PMC11687546 28936944

[120] Bonfante P, Genre A. Mechanisms underlying beneficial plant–fungus interactions in mycorrhizal symbiosis. *Nat Commun* 2010;1:1046. 10.1038/ncomms1046 20975705

[121] Mei Y, Zhang M, Cao G. et al. Endofungal bacteria and ectomycorrhizal fungi synergistically promote the absorption of organic phosphorus in *Pinus massoniana*. *Plant Cell Environ* 2023;47:600–10. 10.1111/pce.14742 37885374

[122] Zhang A-Y, Zhang M-L, Zhu J-L. et al. Endofungal bacterial microbiota promotes the absorption of chelated inorganic phosphorus by host pine through the ectomycorrhizal system. *Microbiol Spectr* 2023;11:e00162–23. 10.1128/spectrum.00162-23 37404161 PMC10433794

[123] Pakvaz S, Soltani J. Endohyphal bacteria from fungal endophytes of the Mediterranean cypress (*Cupressus sempervirens*) exhibit *in vitro* bioactivity. *For Pathol* 2016;46:569–81. 10.1111/efp.12274

[124] Bai H-Y, Zhang A-Y, Mei Y. et al. Effects of ectomycorrhizal fungus bolete identity on the community assemblages of endofungal bacteria. *Environ Microbiol Rep* 2021;13:852–61. 10.1111/1758-2229.13007 34494716

[125] Bretschneider T, Heim JB, Heine D. et al. Vinylogous chain branching catalysed by a dedicated polyketide synthase module. *Nature* 2013;502:124–8. 10.1038/nature12588 24048471

[126] Kusebauch B, Busch B, Scherlach K. et al. Polyketide-chain branching by an enzymatic Michael addition. *Angew Chem Int Ed* 2009;48:5001–4. 10.1002/anie.200900277 19266509

[127] Chen J, Wei Z, Wang Y. et al. Fumonisin B(1): mechanisms of toxicity and biological detoxification progress in animals. *Food Chem Toxicol* 2021;149:111977. 10.1016/j.fct.2021.111977 33428988

[128] Rahayu G, Maulana I, Widodo I. Endobacterial symbiont of *Fusarium oxysporum* f.sp. cubense and the pathogenicity of their symbiosis towards banana plantling. *IOP Conf Ser: Earth Environ Sci* 2020;457:012051. 10.1088/1755-1315/457/1/012051

[129] Han B-Z, Rombouts FM, Nout MJR. A Chinese fermented soybean food. *Int J Food Microbiol* 2001;65:1–10. 10.1016/S0168-1605(00)00523-7 11322691

[130] Dolatabadi S, Scherlach K, Figge M. et al. Food preparation with mucoralean fungi: a potential biosafety issue? *Fungal Biol* 2016;120:393–401. 10.1016/j.funbio.2015.12.001 26895868

[131] Rohm B, Scherlach K, Mobius N. et al. Toxin production by bacterial endosymbionts of a *Rhizopus microsporus* strain used for ternpe/sufu processing. *Int J Food Microbiol* 2010;136:368–71. 10.1016/j.ijfoodmicro.2009.10.010 19942312

[132] Steyn PS, Tuinman AA, van Heerden FR. et al. The isolation, structure, and absolute configuration of the mycotoxin, rhizonin a, a novel cyclic heptapeptide containing N-methyl-3-(3-furyl)alanine, produced by *Rhizopus microsporus*. *J Chem Soc Chem Commun* 1983;1:47–9. 10.1039/C39830000047

[133] Wilson T, Rabie CJ, Fincham JE. et al. Toxicity of rhizonin a, isolated from *Rhizopus microsporus*, in laboratory animals. *Food Chem Toxicol* 1984;22:275–81. 10.1016/0278-6915(84)90006-1 6539275

[134] Ehinger FJ, Niehs SP, Dose B. et al. Analysis of rhizonin biosynthesis reveals origin of pharmacophoric furylalanine moieties in diverse cyclopeptides. *Angew Chem Int Ed* 2023;62:e202308540. 10.1002/anie.202308540 37650335

[135] Almeida C, Silva Pereira C, Gonzalez-Menendez V. et al. Unveiling concealed functions of endosymbiotic bacteria harbored in the ascomycete *Stachylidium bicolor*. *Appl Environ Microbiol* 2018;84:e00660–18. 10.1128/AEM.00660-18 29858203 PMC6052265

[136] Almeida C, Maddah FE, Kehraus S. et al. Endolides A and B, vasopressin and serotonin-receptor interacting N-methylated peptides from the sponge-derived fungus *Stachylidium* sp. *Org Lett* 2016;18:528–31. 10.1021/acs.orglett.5b03553 26771858

[137] Ibrahim AS, Gebremariam T, Liu M. et al. Bacterial endosymbiosis is widely present among Zygomycetes but does not contribute to the pathogenesis of mucormycosis. *J Infect Dis* 2008;198:1083–90. 10.1086/591461 18694335 PMC2729545

[138] Partida-Martinez LP, Bandemer S, Ruchel R. et al. Lack of evidence of endosymbiotic toxin-producing bacteria in clinical *Rhizopus* isolates. *Mycoses* 2008;51:266–9. 10.1111/j.1439-0507.2007.01477.x 18399908

[139] Yang S, Anikst V, Adamson PC. Endofungal *Mycetohabitans rhizoxinica* bacteremia associated with *Rhizopus microsporus* respiratory tract infection. *Emerg Infect Dis* 2022;28:2091–5. 10.3201/eid2810.220507 36148964 PMC9514336

[140] Tansarli GS, Eschbacher J, Schroeder LK. et al. *Mycetohabitans rhizoxinica* in patients with rhinocerebral mucormycosis due to *Rhizopus microsporus*. *Mycopathologia* 2023;188:151–3. 10.1007/s11046-023-00707-3 36645569

[141] Sephton-Clark P, Muñoz FJ, Itabangi H. et al. Bacterial endosymbionts influence fungal transcriptional profiles with implications for host response in the human fungal pathogens *Rhizopus microsporus* and *Rhizopus delemar*. *bioRxiv* 2020. 10.1101/580746

[142] Huss M, Wieczorek H. Inhibitors of V-ATPases: old and new players. *J Exp Biol* 2009;212:341–6. 10.1242/jeb.024067 19151208

[143] Dekker KA, Aiello RJ, Hirai H. et al. Novel lactone compounds from *Mortierella verticillata* that induce the human low density lipoprotein receptor gene: fermentation, isolation, structural elucidation and biological activities. *J Antibiot* 1998;51:14–20. 10.7164/antibiotics.51.14 9531982

[144] Wurlitzer JM, Stanišić A, Wasmuth I. et al. Bacterial-like nonribosomal peptide synthetases produce cyclopeptides in the zygomycetous fungus *Mortierella alpina*. *Appl Environ Microbiol* 2021;87:e02051–20. 10.1128/AEM.02051-20 33158886 PMC7848919

[145] Wurlitzer JM, Stanišić A, Ziethe S. et al. Macrophage-targeting oligopeptides from *Mortierella alpina*. *Chem Sci* 2022;13:9091–101. 10.1039/D2SC00860B 36091214 PMC9365243

[146] Büttner H, Rassbach J, Schultz C. et al. Beneficial soil fungus kills predatory nematodes with dehydro-peptides translocating into the animal gut. *J Am Chem Soc* 2024;146:34702–10. 10.1021/jacs.4c12989 39652677 PMC11664578

[147] Mondo SJ, Toomer KH, Morton JB. et al. Evolutionary stability in a 400-million-year-old heritable facultative mutualism. *Evol.* 2012;66:2564–76. 10.1111/j.1558-5646.2012.01611.x 22834753

[148] Strullu-Derrien C, Kenrick P, Goral T. et al. Testate amoebae in the 407-million-year-old Rhynie Chert. *Curr Biol* 2019;29:461–467.e462. 10.1016/j.cub.2018.12.009 30661795

[149] Poinar GO . The Evolutionary History of Nematodes as Revealed in Stone, Amber and Mummies, Vol. 9. Leiden, The Netherlands: Koninklijke Brill NV, 2011.

[150] Radosa S, Hillmann F. Host-pathogen interactions: lessons from phagocytic predation on fungi. *Curr Opin Microbiol* 2021;62:38–44. 10.1016/j.mib.2021.04.010 34051610

[151] Price CTD, Hanford HE, Al-Quadan T. et al. Amoebae as training grounds for microbial pathogens. *mBio* 2024;15:e00827–4. 10.1128/mbio.00827-24 38975782 PMC11323580

[152] Salah IB, Ghigo E, Drancourt M. Free-living amoebae, a training field for macrophage resistance of mycobacteria. *Clin Microbiol Infect* 2009;15:894–905. 10.1111/j.1469-0691.2009.03011.x 19845701

[153] Molmeret M, Horn M, Wagner M. et al. Amoebae as training grounds for intracellular bacterial pathogens. *Appl Environ Microbiol* 2005;71:20–8. 10.1128/AEM.71.1.20-28.2005 15640165 PMC544274

[154] Verdú M, Alcántara JM, Navarro-Cano JA. et al. Transitivity and intransitivity in soil bacterial networks. *ISME J* 2023;17:2135–9. 10.1038/s41396-023-01540-8 37857708 PMC10689798

